# Expression of Genes Related to Germ Cell Lineage and Pluripotency in Single Cells and Colonies of Human Adult Germ Stem Cells

**DOI:** 10.1155/2016/8582526

**Published:** 2015-11-08

**Authors:** Sabine Conrad, Hossein Azizi, Maryam Hatami, Mikael Kubista, Michael Bonin, Jörg Hennenlotter, Karl-Dietrich Sievert, Thomas Skutella

**Affiliations:** ^1^Sabine Conrad, P.O. Box 12 43, 72072 Tübingen, Germany; ^2^Institute for Anatomy and Cell Biology, Medical Faculty, University of Heidelberg, Im Neuenheimer Feld 307, 69120 Heidelberg, Germany; ^3^Department of Stem Cells and Developmental Biology, Cell Science Research Center, Royan Institute for Stem Cell Biology and Technology, P.O. Box 19395, Tehran 4644, Iran; ^4^Faculty of Biotechnology, Amol University of Special Modern Technologies, P.O. Box 46168, Amol 49767, Iran; ^5^TATAA Biocenter AB, Odinsgatan 28, 41103 Göteborg, Sweden; ^6^Institute of Biotechnology at the Czech Academy of Sciences Videnska 1083, 14220 Prague 4, Czech Republic; ^7^Institute of Anthropology and Human Genetics, Microarray Facility, University Clinic, Calwerstraße 7, 72076 Tübingen, Germany; ^8^Department of Urology, University of Tübingen Hospital, Hoppe-Seyler-Straße 3, 72076 Tübingen, Germany

## Abstract

The aim of this study was to elucidate the molecular status of single human adult germ stem cells (haGSCs) and haGSC colonies, which spontaneously developed from the CD49f MACS and matrix- (collagen−/laminin+ binding-) selected fraction of enriched spermatogonia. Single-cell transcriptional profiling by Fluidigm BioMark system of a long-term cultured haGSCs cluster in comparison to human embryonic stem cells (hESCs) and human fibroblasts (hFibs) revealed that haGSCs showed a characteristic germ- and pluripotency-associated gene expression profile with some similarities to hESCs and with a significant distinction from somatic hFibs. Genome-wide comparisons with microarray analysis confirmed that different haGSC colonies exhibited gene expression heterogeneity with more or less pluripotency. The results of this study confirm that haGSCs are adult stem cells with a specific molecular gene expression profile *in vitro*, related but not identical to true pluripotent stem cells. Under ES-cell conditions haGSC colonies could be selected and maintained in a partial pluripotent state at the molecular level, which may be related to their cell plasticity and potential to differentiate into cells of all germ layers.

## 1. Background

Human adult germ stem cells (haGSCs) derived from highly enriched spermatogonia isolated from adult human testicular tissue were shown to be highly versatile and having some similarities with human embryonic stem cells (hESCs), including the expression of genes associated with pluripotent cells and the ability to be* in vitro* differentiated into a number of cell lineages comprising the three germ layers [1–6].

In the studies of Mizrak et al. [5], Chikhovskaya et al. [7], and Gonzalez et al. [8], the cells expressing markers of pluripotency were probably derived from mesenchymal stem cells (MSCs) or were more MSC-like. Moreover, it has also been proposed that haGSCs may be low-differentiated testicular fibroblasts [9]. In contrast, Stimpfel et al. [10] demonstrated that both germ- and mesenchyme-derived stem cells were present in stem cell clusters from human testis biopsy, which could differentiate into cells of all three germ layers. Recently, Lim et al. [6] provided evidence that haGSCs show similarities to hESCs and are being able to generate small teratomas.

The findings of all these studies raised some new questions about the real character of pluripotency in haGSCs. It is generally accepted that pluripotency of cells requires the activation of a transcriptional regulatory network [11], a phenomenon which has been observed in* ex vivo* cultures of early embryonic cells and also in cells of the germ cell lineage, in which members of the pluripotency network are normally active, including embryonic cells during development of morula and blastocyst-stage (inner cell mass) embryo, epiblast, primordial germ cells (PGCs), and germline stem cells.

One main step in analyzing the biology of haGSCs and pluripotency in adult stem cells is to determine their germ cell-specific gene expression profile. The present knowledge regarding the molecular markers that define haGSCs and their pluripotency is significantly limited. Therefore, the goal of this study was to investigate the molecular profile of haGSCs, which are able to comprise both the expression of a residual germ cell profile and genes related to pluripotency, in addition to our previous study on hSSCs [1]. In order to accomplish this goal, we sought to compare the gene expression profiles of haGSCs generated from short-term cultured enriched spermatogonial stem cells (hSSCs) to hFibs and hESCs using (1) single cell nanofluid real-time PCR (Fluidigm) of a representative haGSC colony, (2) microarray analysis, and (3) Fluidigm real-time PCR and immunohistochemistry of haGSC colonies to validate the microarray data. Here, we show that haGSCs are adult stem cells with a specific molecular profile, which is related to spermatogonia. Under hESC culture conditions they can be selected and cultured and maintain a state resembling in part gene expression related to the expression patterns found in pluripotent cells.

## 2. Results

### 2.1. Generation of haGSC Colonies from Enriched Fraction of Spermatogonia

**Figure 1:**
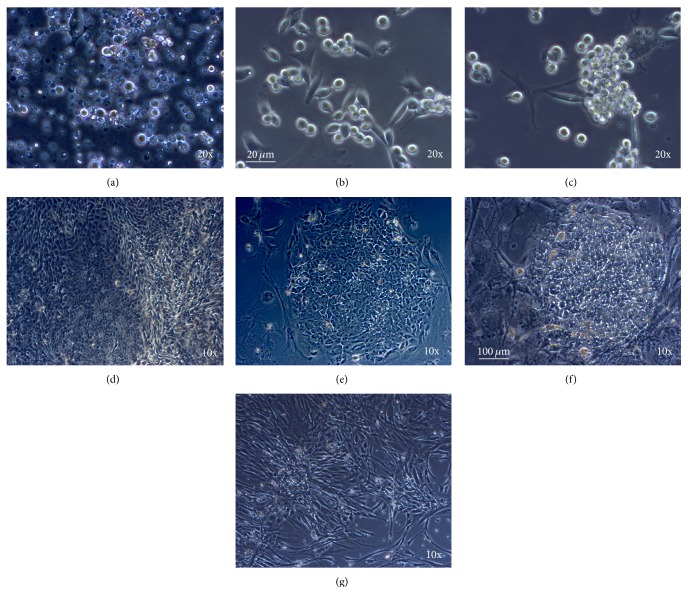
haGSC colonies were derived from enriched spermatogonia and share morphological similarities with early hESC colonies after passaging. Typical representative morphology of spermatogonia and haGSCs from the same patient (157) during culture. (a) Human testis dissociated cells during the first week of culture before selection of spermatogonia. ((b) and (c)) Single, pairs, chains, and groups of round cells with high nucleus/cytoplasm ratio typical of spermatogonia were observable after selection. (d) After 4–6 weeks, some aggregations of the first epithelial haGSCs were observed. (e) Examples of typical early haGSC, (f) early hESC colonies, and (g) typical fibroblasts from testis. Scale bar in ((a)–(c)) 20 *μ*m and ((d)–(g)) 100 *μ*m.

Colonies or clusters of haGSC developed spontaneously from the CD49f MACS and matrix (collagen nonbinding, laminin binding) selected fraction of enriched spermatogonia (Figure 1) but not from the negative selected fraction of cells or from patients without spermatogonia. By MACS and matrix selection, the hFibs, which overgrow the primary cell cultures, were depleted and remained in the nonselected populations of cells. The hFibs appeared morphologically completely different compared to haGSCs (Figure 1). In the primary cultures, the first small haGSC colonies/islands started to appear 4–6 weeks after culture of enriched spermatogonia in hGSC medium. The denser haGSC aggregations were manually selected for further propagation and characterization (Figure 1(d)). The typical haGSC colony consisted of central part of colony and outgrowing epithelial cells resembling early cell colonies of hESCs (Figure 1(e)). In the negatively selected cell fraction, no epithelial haGSC colony formation was observed [9]. This typical epithelial morphology is an important distinction to hFibs (Figure 1(g)).

### 2.2. Single Cell Analysis of hFibs, hESCs and haGSCs

**Figure 2:**
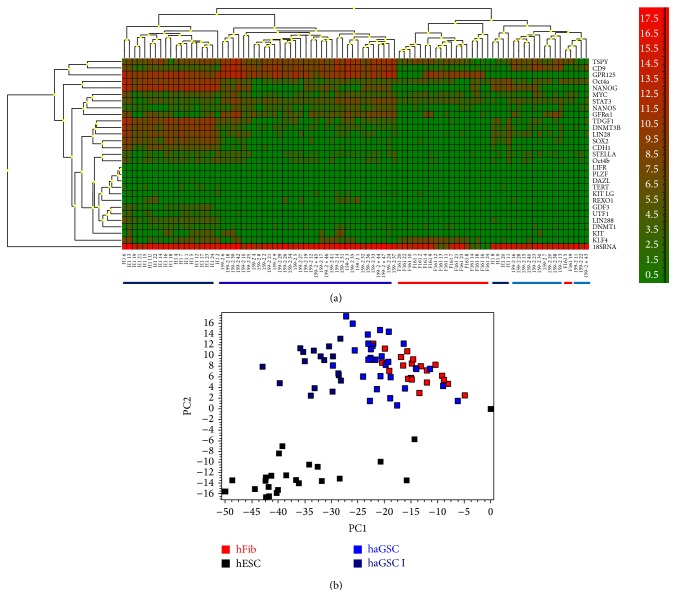
Gene expression profiling of single hESCs, haGSC, and hFibs of germ cell-enriched and pluripotency associated genes. (a) Heat map showing array of pluripotency and germ cell associated genes with each column representing a single cell. Note that haGSCs (coloured blue) are a heterogenic population of cells with more similarities to hESCs (coloured black), while some outliner cluster with hFibs (coloured red). (b) PCA showing the distribution of single cells selected from the separated trees from (a). The group of haGSCs which cluster with hESCs in (a) are named haGSCs I and were coloured dark blue.

**Figure 3:**
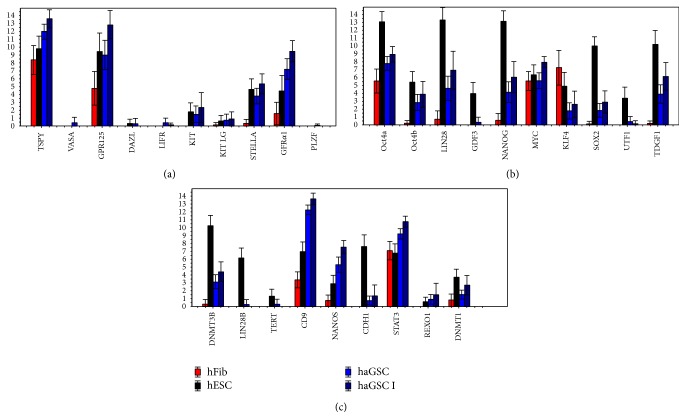
Bar plot showing relative expressions of (a) germ- and (b, c) pluripotency-associated genes between haGSCs (coloured light blue), haGSC I (coloured dark blue), hESCs (coloured black), and hFibs (coloured red). For *P* < 0.05 haGSCs versus hFibs and *P* < 0.05 haGSC I versus hFibs, see Supplementary Table 3.

As can be seen from the dendrogram in Figure 2(a), the nanofluid single cell real-time PCR gene expression profiling from a typical single haGSC colony revealed that most cells from the group of haGSCs (72.3%, 34/47 cells) clustered together in a single tree, most closely related to hESCs, where also most of the cells clustered in a single tree (82.6%, 19/23 cells). These two groups were separated from hFibs which clustered in a single tree with several outliner haGSCs (26.7%, 13/47 cells) and a few outliner hESCs (17.4%, 4/23 cells). The majority of single haGSCs clearly separated from hFibs expressing genes of pluripotency, combined with a residual germ cell profile distinct from hFibs (Figures 2 and 3). According to the dendrogram, the haGSCs were divided into two groups: the group of all haGSCs and the group of haGSCs without outliners (group I) for further analysis (Figure 2(b)).

When analysing all the selected germ- and pluripotency-associated genes in more detail, the gene expression profiling showed that the group of all haGSCs expressed a high level of the known germ cell-specific genes* STELLA*, the GDNF-receptor* GFRα1*,* TSPYL,* and* CD9* (haGSCs versus hFibs, *t*-test and Mann-Whitney test, *P* < 0.05) (Figure 3). In this single cell analysis, the germ cell associated genes* VASA*,* DAZL*, and* LIFR* were not expressed by any group of cells. Furthermore, haGSCs expressed the pluripotency-related genes* NANOS, DNMT3B, TDGF1, STAT3, NANOG, LIN28, GPR125, OCT4A*, and* SOX2* at a significantly higher level than hFibs, while hFibs expressed* KLF4* at a higher level than haGSCs, as revealed by *t*-test and Mann-Whitney test (*P* < 0.05) (Figure 3, Supplementary Table 3, available online at http://dx.doi.org/10.1155/2016/8582526). In the comparison of haGSC group I and hFibs, the same array group of genes and* DNMT1* were significantly regulated in haGSCs, although at a higher intensity (Figure 3 and Supplementary Table 3). The hFibs did not show any amplification product for* SOX2, UTF1, TDGF1, LIN28B, TERT, *and* CADH1*.

When comparing single hESCs with haGSCs group I (Figure 3 and Supplementary Table 3), hESCs more strongly expressed most of the pluripotency-related genes, including* SOX2, NANOG, LIN28, LIN28B, GDF3, CADH1, OCT4a, TDGF1,* and* UTF1*, while haGSCs more strongly expressed the germ cell-related genes* CD9, GFRα1, NANOS, STAT3, TSPYL, GPR125,* and* MYC* (hESCs versus haGSCs group1, *t*-test and Mann-Whitney test, *P* < 0.05) (Supplementary Table 3). The analysis of all haGSCs revealed a similar profile with lower expression levels (Figure 3 and Supplementary Table 3). This indicates that the haGSCs expressed the core pluripotency-related genes including* OCT4a*,* NANOG*,* SOX2,* and* LIN28*, while still retaining a partial germ cell-related gene expression profile. The intensity of the expression of the core pluripotency-related genes was lower in haGSCs than in hESCs but significantly higher than in hFibs (Supplementary Table 3).

On a single cell level haGSCs are a heterogeneous population of cells, where there are 12 out of 47 (25.5%) coexpressed transcripts of core pluripotency genes* OCT4a, NANOG, *and* SOX2* (Figure 2(a)).

These observations encouraged us to have a closer look at the changes in genes global expression and in particular the expression of germ-, pluripotency-, fibroblast-, and mesenchymal-associated genes and genes activated during ESC-like haGSC cluster formation (natural reprogramming of human spermatogonia).

### 2.3. Microarray Gene Expression Profiling

To this end, we investigated the transcriptome of the following cell types by microarray technology 4 cell groups: hFibs (F161; negative control), hESCs (H1; positive control), short-term cultured hSSCs, and 4 different natural reprogramming trials of single clones of haGSCs.

#### 2.3.1. Sample to Sample Relations

**Figure 4:**
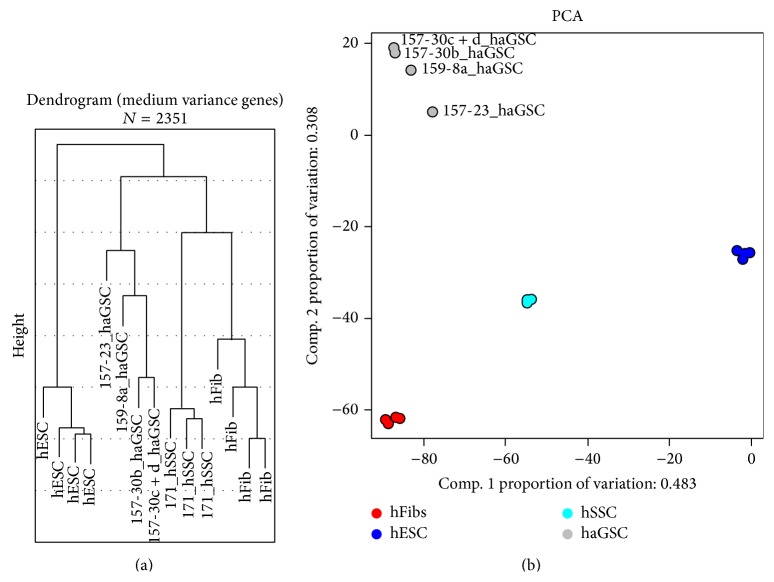
(a) Dendrogram using only genes with median variance. Genes with median variance were defined by selecting all genes whose variance is at least 2 SDs away from mean variance (2357 genes). Dendrograms were calculated using average linkage clustering and Euclidean distance. (b) Scatter plot of the first two principle components. The first two components explain 79% of the variance.

In the first step, we investigated relations between samples especially with regard to different cell types and reprogramming trials. To this end, hierarchical clustering (complete linkage with Euclidean distance) and as well as a principal component analysis (PCA) were performed (Figure 4). For both methods, the set of genes was reduced to the medium variance genes (gene whose standard deviation is higher than the average standard deviation + 2SDs). All the different cell types were clearly separated by the two best principal components, which explain 79% of the complete variance. The same separation was observed in the cluster dendrogram. In particular, hESCs and hSSCs were aggregated in nicely separated clusters. However, the group of haGSCs was more heterogeneous and the sample 157-23_P5 especially seemed to be more different. The cluster dendrogram was very similar even when the whole transcriptome (all 54K genes) was considered (data not shown).

Then we further investigated the Pearson correlation of the 4 distinct haGSCs to the three other cell types based on the medium variance genes. Three out of the four haGSCs showed their highest correlation to hSSCs with the Pearson correlation coefficient of 0.82–0.85 while the correlation coefficient to hESCs and hFibs was lower between 0.75 and 0.79. Only reprogramming sample 157-30c+d showed the highest correlation coefficient to hFibs.

This analysis clearly pointed that all cell types showed a distinct gene expression pattern and they were clearly distinguishable from each other. This was true for the selected set of genes based on the medium variance genes but was also found for the complete transcriptome.

#### 2.3.2. High Variance Genes

**Figure 5:**
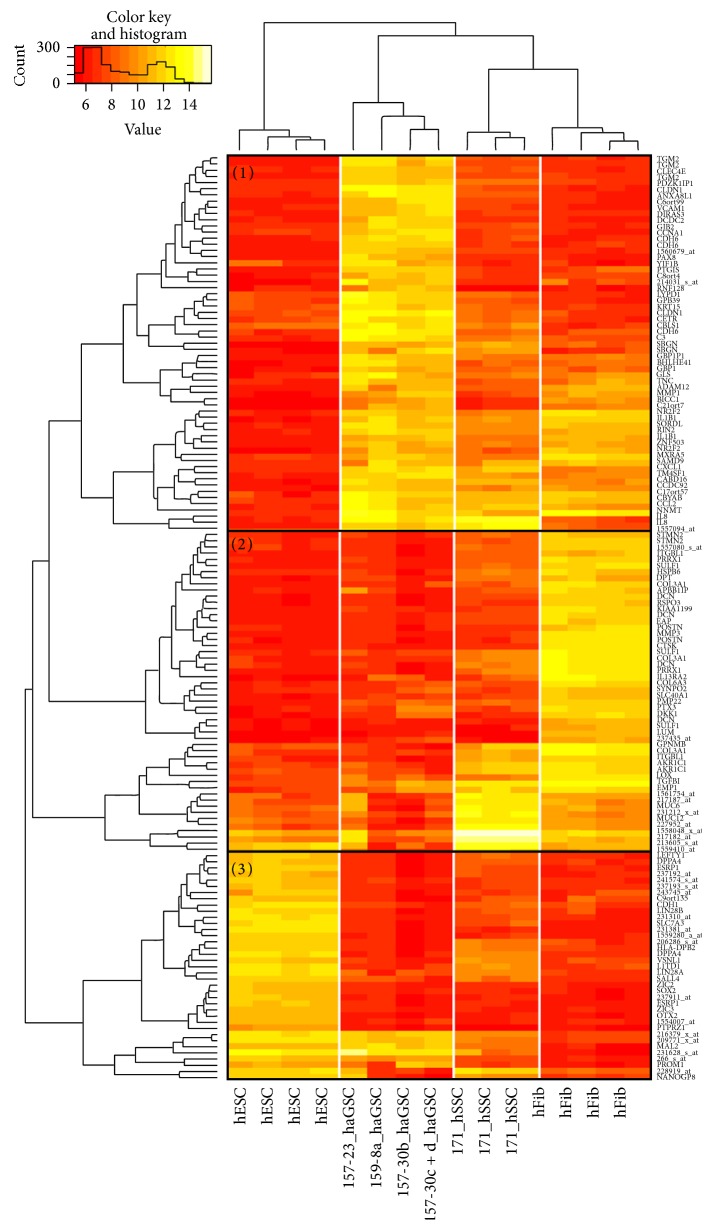
Heatmap of 150 features with the highest variance generated by independent clustering of genes and samples using complete linkage clustering and Euclidean distance. All cell types were separated consistently and grouped correctly into subtrees. Each cell type was characterized by a specific set of highly expressed genes (block pattern). The set of high variance genes could be grouped in 3 classes of genes with high expression: (1) in hESCs (H1), (2) in hFibs, and (3) in haGSCs.

For 150 genes with the highest variances across all samples, we calculated a heatmap (independent clustering of samples and genes) (Figure 5). As already seen in the dendrogram of the median variance genes, all cell types were separated consistently and grouped correctly into subtrees. The high variance genes were clustered in cell type-specific sets of highly expressed genes (visible as block pattern). The set of high variance genes could be grouped in 3 classes of genes with high expression: (1) in haGSCs, (2) in hFibs, and (3) in both hESCs and haGSCs.

Each of the three groups of genes was searched for enrichment of gene ontology (GO) terms. The 10 most significant GO terms for each group are shown in Supplementary Table 4. Genes highly expressed in hFibs showed typical functional annotation, which involves mainly extracellular matrix production, including collagen and proteoglycan metabolic process. The hESC genes were typically related to embryonic morphogenesis/development and diverse positive regulated cellular processes. In partial overlap with the hESC group, hSSCs and haGSCs expressed several hESC cell-related genes, including* NANOG*,* LIN28,* and* SALL4*. The genes which were highly expressed in haGSCs revealed a heterogeneous enrichment of gene ontology terms including biochemical processes, which partially overlapped with hSSC and hFib mechanisms of cell adhesion and immunological defense responses (Supplementary Table 4).

#### 2.3.3. Analysis of haGSCs with Predefined Gene Sets for Germ-, Pluripotency-, Fibroblast-, and MSC-Associated Genes from the Literature

In an extended approach, we considered different predefined sets of genes: (1) ES cell-specific genes, a gene set containing human pluripotency-related genes specific for ES cells, and (2) hSSC-specific genes, a set of genes specific for germ cells. These two sets reflected the general knowledge from the literature. In addition to these two sets of genes, we used (3) genes which were found to be related to mesenchymal stem cells (MSCs) according to Chikhovskaya et al. [7], (4) hESC-enriched genes, and (5) genes found to be enriched in hFibs. The last two sets of genes (4 and 5) were extracted from the publication of Ko et al. [9] (Figure 1k -Human ES cell-enriched genes). The expression of three different sets of genes 1, 2, and 3 is presented in heatmaps in Supplementary Figures 1 and 2.

The analysis clearly showed that (1) none of the hESC cell-specific genes were expressed in hFibs. The genes* PROM1*,* FOXO1*, and* CD24* had a higher expression in haGSCs and in one of them (157-23_P5) also the genes related to pluripotency* NANOG*,* LIN28A*,* SALL4,* and* POU5F1* were expressed at a higher level than in hFibs. In contrast, the microarray analysis revealed that some genes were highly expressed in hESCs but not in “reprogramming trials” (*SOX2*,* ZIC3*,* DPPA4*, and* LEFTY2*). (2) From the set of hSSC-specific genes,* GFRα1*,* TSPYL*,* GPR125*, and* CD9* showed a higher expression in haGSCs than in hFibs. Some of the MSC-related markers, which are commonly used for the characterization of MSCs, are CD73, CD29, CD90, CD105, CD140b, CD146, and CD166. (3) Most of the set of MSC-related genes which could be detected by microarray analysis (*CD73*,* CD29*,* CD90*,* CD105*,* CD146*, and* CD166*) were not differentially expressed in haGSCs in comparison to hFibs or hESCs (see heatmap, Supplementary Figure 2). The gene* CD146* (*MCAM*) which was used to strengthen the similarity of testicular stem cells to MSCs by Chikhovskaya et al. [7] was also not differentially expressed in haGSCs in comparison to hFibs and hESCs. Furthermore, most of the genes used by Chikhovskaya et al. [7] to demonstrate that their testis-derived stem cells might be MSC-like were also not differentially expressed in haGSCs in comparison to hFibs and hESCs. Additionally, also the genes* CD34* and* CD73* which were used by Choi et al. [12] to isolate their testis-derived fibroblasts were not expressed in haGSCs. In our analysis, the only MSC-specific genes which were differentially expressed in haGSCs were* VCAM1*,* FN1*,* CDCP1*,* TM4SF1,* and* IL6ST*. There were also some other MSC-specific genes such as* NCAM1*,* CD44,* and* BMPR2,* which were expressed in hSSCs or hESCs. A heatmap for the expression of genes for gene sets is shown in Supplementary Figure 2 which corresponded to Figure 1k from the publication of Ko et al. [9]. From this analysis, it was evident that some ESC-enriched genes, like* TERF1*,* CXADR*,* PHC1*,* HOOK1*,* NANOG*,* POU5F1*,* SALL4*, and* LITD1*, were highly expressed in “reprogramming” trials, while others were not. A similar situation was observed for the set of hFibs-enriched genes. Some of them (*NNMT*,* IL1R1*,* NR2F2*, and* GREM1*) were highly expressed in haGSCs, while others did not show any regulation.

Considering the Pearson correlation based on five gene sets, three out of the four haGSC samples showed similar correlation coefficients of 0.48–0.55 to hFibs and to hESCs. But, one “reprogramming” sample (157-23_P5) showed a higher correlation to hESCs (0.68) and lower correlation to hFibs (0.46). Of the four “reprogramming” samples, this sample showed the highest similarity to hESCs.

The expression profiling data support the conclusion that haGSCs were not hFibs or MCSs but instead possess a strong germ-line specific background and a degree of similarity to hESCs.

#### 2.3.4. Extended Profile Search

The differential analysis comparing haGSC samples with hFibs and hESCs allowed to identify differentially expressed genes in these groups of cells. Our special attention went to the comparison of “reprogramming” sample 157-23_P5 to hFibs and hESCs. The haGSC sample 157-23_P5 was selected since it showed the highest similarities to hESCs by other comparisons.

**Figure 6:**
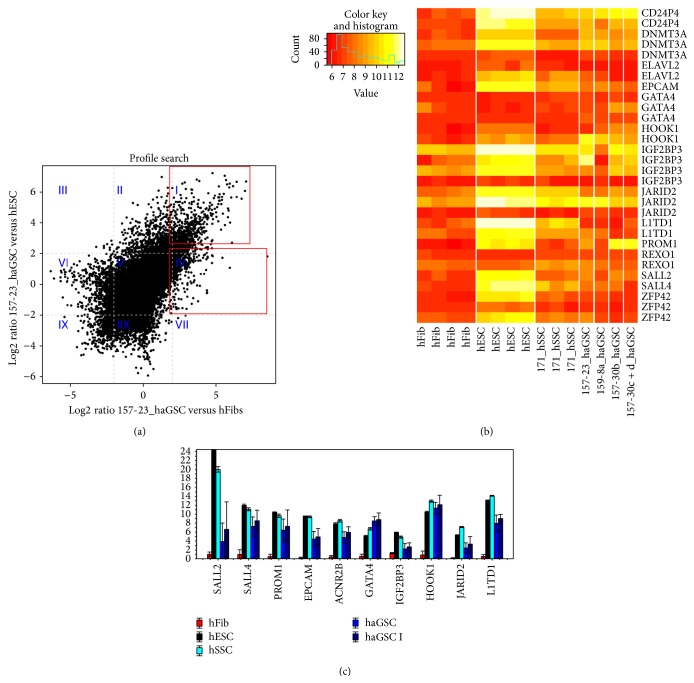
(a): Scatter plot of log_2_⁡ ratios for the two differential analyses of reprogramming versus hFibs (*x*-axis) and haGSCs versus hESCs (*y*-axis). Grey dashed lines represent the thresholds of −2 and 2 used definition of the sectors. Different sectors are labelled with blue-coloured Roman numerals. The plot is divided into nine different sectors taking log_2_⁡ ratios of −2 and 2 as thresholds which corresponds to a 4-fold differential regulation. The nine sectors were as follows. Sector I 798 genes were upregulated in 157-23_P5 compared to hFibs and upregulated in 157-23_P5 compared to hESCs. Sector II 895 genes were not differentially regulated in 157-23_P5 compared to hFibs and upregulated in 157-23_P5 compared to hESCs. Sector III 8 genes were downregulated in 157-23_P5 compared to hFibs and upregulated compared to hESCs. Sector IV 454 genes were upregulated in 157-23_P5 compared to hFibs and not differentially regulated in 157-23_P5 compared to hESCs. Sector V 49468 genes are not differentially regulated in 157-23_P5 compared to hFibs and not differentially regulated in 157-23_P5 compared to hESCs. Sector VI 855 genes are downregulated in 157-23_P5 compared to hFibs and not differentially regulated in 157-23_P5 compared to hESCs. Sector VII 2 genes are upregulated in 157-23_P5 compared to hFibs and downregulated in 157-23_P5 compared to hESCs. Sector VIII 1744 genes are not differentially regulated in 157-23_P5 compared to hFibs and downregulated in 157-23_P5 compared to hESCs. Sector IX 451 genes are downregulated in 157-23_P5 compared to hFibs and downregulated in 157-23_P5 compared to hESCs. The majority of the genes were not differentially expressed between 157-23_P5, hFibs, and hESCs (49K gene in Sector V). (b) Heatmap with pluripotency associated genes upregulated by haGSCs in comparison to hFibs according to microarray experiment. (c) Gene expression profiling of hESCs, haGSCs, and hFibs of additional pluripotency associated genes upregulated according to microarray study in haGSCs. (a) haGSCs are a heterogenic population of cells with some similarities to hESCs (coloured black) and hSSCs (coloured aquamarine) but not to hFibs (coloured red).

For comparing 157-23_P5 with hFibs and hESC, log_2_⁡ ratios of the two corresponding differential analyses (comparisons of reprogramming trials with hFibs and with hESCs) were plotted in a scatter plot. The plot was divided into nine different sectors taking log_2_⁡ ratios of −2 and 2 as thresholds which corresponds to a 4-fold differential regulation (see Figure 6(a)).

The majority of genes were not differentially expressed between 157-23_P5, hFibs, and hESCs (49K genes in Sector V). But there were still many genes showing similar expression in 157-23_P5 and hESCs but differential expression between 157-23_P5 and hFibs (454 genes in Sector IV and 855 genes in Sector VI). Considering the expression of these genes, reprogramming sample was similar to hESCs and different from hFibs. On the other hand, there were also many genes, which showed similar expression in both the 157-23_P5 reprogramming sample and hFibs, while they were differentially expressed between 157-23_P5 and hESCs (895 genes in Sector II and 1744 genes in Sector VIII). According to these genes, the reprogramming sample 157-23_P5 was similar to hFibs but different from hESCs. Additionally, 798 genes in Sector I were upregulated in reprogramming sample in comparison to hFibs and hESCs.

For the genes in Sectors II, IV, VI and IIX, a functional annotation was performed. The 10 GO terms with the most significant *P* values are shown in Supplementary Table 4. The genes in Sector VI were expected to be related to fibroblast-enriched GO terms, since these genes showed a high expression in fibroblasts but low expression in the reprogramming sample 157-23_P7. And indeed these genes were related to the “extracellular region” and “matrix” terms. Analogously the genes in Sector VIII showed high expression in hESCs but low expression in the 157-23_P7 sample. These genes were related to hESC cell-linked GO terms such as “nuclear part” or “cell cycle.” The genes, which were upregulated in the sample 157-23_P7 from Sector IV, were mostly linked to GO terms of “nuclear processes.”

#### 2.3.5. IPA Ingenuity Analysis

The Sector I genes (upregulated in the haGSCs sample 157-23_P5 compared to hFibs and hESCs) and Sector IV genes (upregulated in the sample 157-23_P5 compared to hFibs and not differentially regulated in 157-23_P5 compared to hESCs) were analyzed by IPA Ingenuity programme.

The results showed that, among Sector I genes which were upregulated in haGSCs in comparison to both hFibs and hESCs, there were gene networks related to (1) amino acid metabolism, small molecule biochemistry, and gene expression (39 genes), (2) cellular assembly and organization, cellular function and maintenance, skeletal and muscular system development and function (37 genes), (3) cell morphology, cellular assembly and organization, and gastrointestinal disease, and (4) cell-to-cell signaling and interaction, cellular assembly and organization, and cellular function and maintenance (28 genes). Among these genes the most expressed canonical pathways were TREM1 signaling, hepatic fibrosis/hepatic stellate cell activation, ILK signaling, IL-17A signaling in airways cells, and production of nitric oxide and reactive oxygen species in macrophages. The main transcription factors were* RELA*,* NFΚB* (complex),* CTNNB1*,* NFΚBIB*, and* Nfat* (family). In terms of molecular and cellular functions, the highest proportion of upregulated genes was related to cellular growth and proliferation (190 genes), cell death (187 genes), cellular function and maintenance (144 genes), cell morphology (139 genes), and cellular movement (130 genes). In terms of physiological system development and function, the highest proportion of upregulated genes was related to tissue development (180 genes), organismal development (132 genes), tissue morphology (111 genes), cardiovascular system development and function (82 genes), and connective tissue development and function (42 genes). In terms of disease, the highest proportions of upregulated genes were related to cancer (230 genes) and reproductive system disease (133 genes). In comparison to hFibs, the most upregulated genes in haGSCs were* CLDN1*,* LYPD1*,* GPR39*,* PAX8*,* CFTR*,* NCAM1*,* PARD6B*, and* CRLS1*, while the most upregulated genes in comparison to hESCs were* KRT7*,* IL8*,* GBP1*,* C3*,* BHLHE41*,* CLDN1*,* PAX8*, and* CCL2*.

Among Sector IV genes, which were upregulated in haGSCs (157-23_P5) in comparison to hFibs and not differentially regulated in comparison to hESCs, there were gene networks related to (1) RNA posttranscriptional modification, cell morphology, cellular assembly, and organization (57 genes), (2) cellular assembly and organization, cellular compromise, and nervous system development and function (52 genes), (3) gene expression, cellular growth and proliferation, and embryonic development (50 genes), (4) cell-to-cell signaling and interaction, cellular assembly and organization, and tissue development (44 genes), and (5) cancer, hematological disease, DNA replication, recombination, and repair (41 genes). Among these genes the most expressed canonical pathways were Sertoli cell-Sertoli cell junction signaling, tight junction signaling, cell cycle: G1/S checkpoint regulation, Wnt/catenin signaling, and chronic myeloid leukemia signaling. The main transcription regulators were* YY1*,* FOXD3*,* E2F4*,* HOXA9*, and* NPAT*. In terms of molecular and cellular functions, the highest proportion of genes was related to gene expression (83 genes), cellular assembly and organization (46 genes), cellular function and maintenance (34 genes), cell-to-cell signaling and interaction (24 genes), and RNA posttranscriptional modification (23 genes). In terms of physiological system development and function, the highest proportion of genes was related to tissue development (43 genes), nervous system development and function (30 genes), cardiovascular system development and function (28 genes), organ morphology (27 genes), and skeletal and muscular system development and function (7 genes). In terms of disease, the highest proportions of genes were related to cancer (153 genes) and reproductive system disease (76 genes).

Interestingly, among Sector IV genes, which were upregulated in haGSCs (157-23_P5) in comparison to hFibs and not differentially regulated in comparison to hESCs, there was also a network of 50 genes related to gene expression, cellular growth and proliferation, and embryonic development, including the genes related to pluripotency. All these genes were upregulated in the haGSC sample 157-23_P5 in comparison to hFibs. The most upregulated gene in haGSCs was* NANOG*. These results indicated that the haGSC reprogramming sample 157-23_P5 expressed a relatively higher degree of pluripotency genes, which was significantly higher than in hFibs.

#### 2.3.6. Validation of Microarray Results by Real-Time PCR and Immunohistochemistry

In addition to the initial panel of germ cell- and pluripotency-associated genes, the following germ cell- and pluripotency-associated genes were also selected for the Fluidigm real-time PCR analysis (see Figure 6(c) and Supplementary Figure 3) based on microarray results (see Figure 6(b)):* L1TD1*,* SALL4*,* JARID2*,* HOOK1*,* EPCAM*,* PROM1*,* SALL2*,* IGFR2BP3*,* REX1*, and* GATA4*. In similarity to haGSC clones derived from patients 157 and 159, the genes* VASA*,* DAZL*, and* PLZF* were predominantly expressed in haGSCs derived from two additional patients 239 and 240 (Supplementary Figure 3). The haGSCs showed a profound decreased expression of germ cell-specific genes* VASA*,* DAZL*, and* PLZF* in comparison to hSSCs. In contrast, the other two germ cell-specific genes,* STELLA* and* GFRα1*, were strongly expressed in haGSCs. In comparison to hSSCs the genes* REX1*,* LIFR*, and* NANOS* were expressed in haGSCs in a similar range than in hSSCs, while the gene* CD9* was more strongly expressed in haGSCs. The genes* DAZL* and* LIFR* were not expressed in hFibs at all. All germ cell-associated genes were significantly more strongly expressed in hSSCs and haGSCs than in hFibs (*t*-test, <0.05). In comparison to hSSCs, haGSCs similarly compared to hESCs possessed a rudimentary germ cell- associated gene expression profile. The genes* CD9* and* GFRα1* were more strongly expressed in haGSCs than in hESCs (Supplementary Figure 3).

The expression of pluripotency-associated genes was similar in haGSCs clones from two different patients 239 and 240. All pluripotency-associated genes were significantly upregulated in the best haGSC clones in comparison to hFibs (*t*-test, <0.05) (Supplementary Figure 3 and Supplementary Figure 4b). From the genes in Sector IV, which were differentially upregulated in the clone 157-23_P5 according to the previous microarray analysis, 8 pluripotency- and germ cell-associated genes were reconfirmed, with the exception of genes* SALL2* and* IGFR2BP3* (Figure 6(c)).

**Figure 7:**
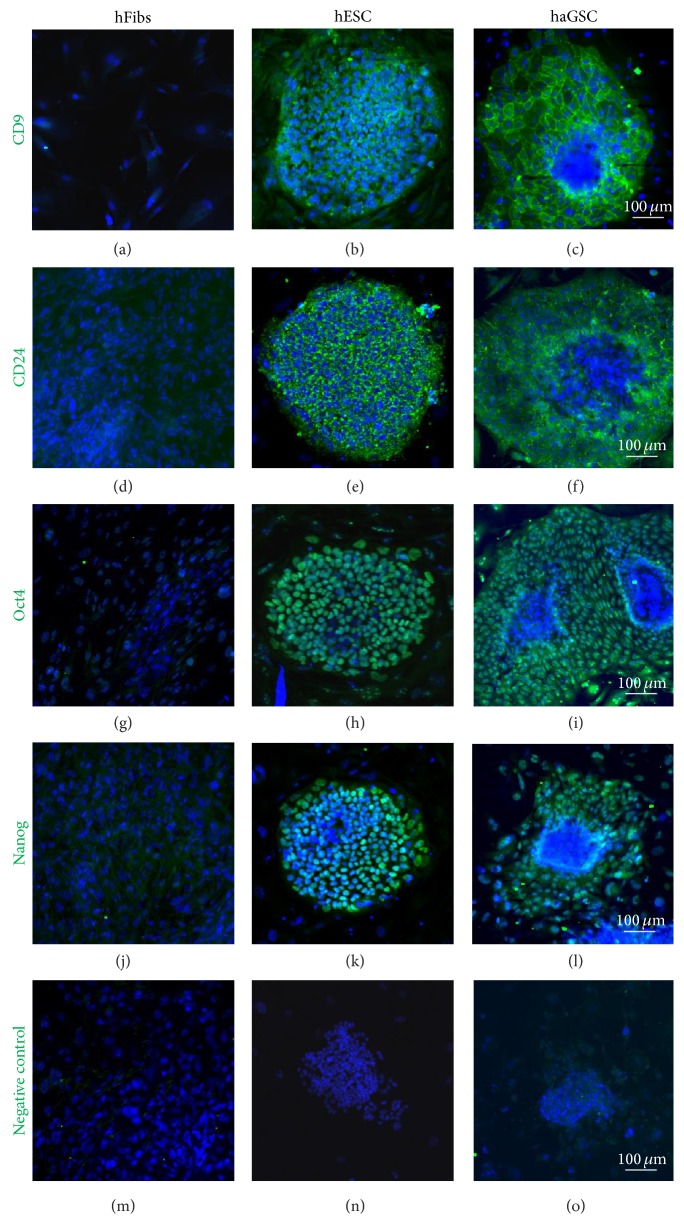
Immunohistochemistry of hESCs, haGSCs, and hFibs with CD9, CD24, Oct4, and Nanog antibodies. The different markers are shown in green and the staining of the nuclei with DAPI in blue. Scale bar is 100 *μ*m.

**Figure 8:**
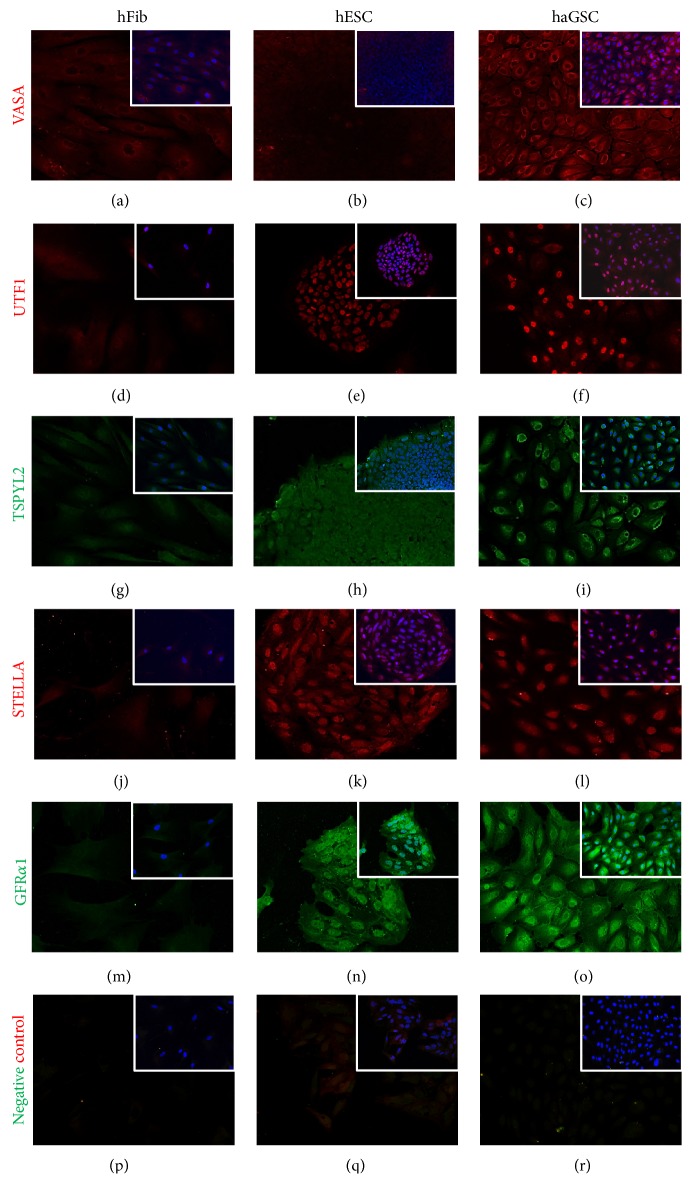
Immunohistochemistry of hFibs, hESCs, and haGSCs with cytoplasmic staining of VASA ((a)–(c)), nuclear staining of UTF1 ((d)–(f)), cytoplasmic staining of TSPYL2 ((g)–(i)), cytoplasmic and nuclear staining of STELLA ((j)–(l)), and cytoplasmic staining of GFR*α*1 ((m)–(o)). The different germ cell different markers are shown in green (Alexa 488 for TSPYL2 and GFR*α*1) or red (Alexa 546 for VASA, UTF1, and STELLA) and the staining of the nuclei with HOECHST is shown in blue. Scale bar is 50 *μ*m.

Immunocytochemistry of haGSC clusters in comparison to hESCs and hFibs clearly demonstrated that CD9, CD24, Oct4, and Nanog were expressed by haGSCs and hESCs but not in hFibs (Figure 7). Furthermore immunohistochemical staining with typical germ cell proteins of haGSC clusters in comparison to hESCs and hFibs also clearly demonstrated that VASA, UTF1, TSPYl2, STELLA, and GFR*α*1 were expressed by haGSCs and in part by hESCs, but not in hFibs (Figure 8).

## 3. Discussion

The haGSCs displayed a specific gene expression profile, with some properties of both germ cells and hESCs. In general, they showed a higher expression of some ESC-related genes, which were not expressed in hFibs in this study. In the microarray study this distinct gene expression profile was found on all three presented levels: (1) the heatmap of high variance genes showed many pluripotency-related genes which were highly-expressed in haGSCs; (2) in particular one out of four reprogramming samples of haGSCs (157-23_P5) showed a higher similarity to hESCs. However, none of the reprogramming samples exhibited a full pluripotency-related gene expression profile, but only to a degree, as reported by some other groups [13].

According to the microarray experiments in this study, the core pluripotency-related genes* NANOG*,* LIN28*, and* POU5F1* were upregulated in haGSCs in comparison to hFibs, while* SOX2* was not. As revealed by pilot single-cell Fluidigm analysis and also by a reconfirmation experiment with new haGSC clones by the same technology, haGSCs expressed all above mentioned pluripotency-associated genes, including* Sox2, TDGFβ1*, and* UTF1*, and also some germline-associated genes, such as* STELLA*,* CD9*,* GFRα1*, and* TSPY*.

As published by some other groups, the list of significantly regulated genes in haGSCs in comparison to hFibs included several genes which are highly expressed in hESCs, such as* CD24* [14],* EPCAM* [15–18],* L1TD1* [19, 20],* SALL4* [21],* JARID2* [22–24],* DNMT3A* [25, 26],* HOOK1* [27],* ACTIVIN A receptor 1B* [28], and* REX1* [29, 30]. The significant upregulation of these genes in haGSCs, revealed by microarray analyses, was also reconfirmed by Fluidigm RT-PCR and for CD24 by immunohistochemistry.

It is important that there were several genes related to pluripotency, which were regulated in haGSCs. The microarray analysis showed that the cell surface protein-encoding gene* CD24* was strongly expressed in all haGSC clones and to a lesser extent also in the enriched population of spermatogonia. The gene* CD24* was identified as one of the hESC-associated genes proposed by Assou et al. [14] by carrying out a meta-analysis of the hESC transcriptome. This gene encodes a membrane-specific protein, which is strongly expressed in hESCs and enables purifying them by FACS from cocultured fibroblasts.

In this study, we also found that* CD9* and* EPCAM* were also strongly expressed in both haGSCs and hESCs. It is known that the pluripotency-associated epithelial surface marker* EPCAM* is strongly expressed in human fetal gonads and can be effectively used as a selector marker for germ cell enrichment from differentiating ES cells [18]. This molecule has been shown to play a role in progenitor proliferation in the mouse SSC culture system [15]. The gene* EPCAM*, which forms functional complexes with some other genes such as* CLDN7*,* CD44V6*,* TSPAN8*, and* CD9*, might increase the efficiency of transcriptions-factor mediated pluripotency reprogramming by upregulation of* OCT4* and suppression of the p53-p21 pathway [17].

It has already been illustrated that the RNA-binding protein L1TD1, which is highly expressed in haGSCs, is a marker for undifferentiated hESCs [19, 31]. The* L1TD1* gene is also rapidly activated during iPSC generation, but it is dispensable for the maintenance and induction of pluripotency [20]. In these publications it is documented that* LITD1* interacts with the core pluripotency gene* LIN28* and has an important function in the regulation of stemness, including hESC self-renewal and cancer cell proliferation. The* L1TD1* gene is a downstream target of* NANOG* and represents a useful marker to identify undifferentiated hESCs. Additionally, the* L1TD1* gene is also highly expressed in testicular seminoma, and depletion of* L1TD1* in seminoma cancer cells influences their self-renewal and proliferation [20]. One of genes highly upregulated in haGSCs was also* SALL4*. Hobbs et al. [32] have demonstrated the critical and distinct roles of* SALL4* in development of germ cells during embryonic period of life and differentiation of postnatal spermatogonial progenitor cells. It has further been demonstrated that the stem cell-associated gene* SALL4* suppresses the transcription through recruitment of DNA methyltransferases [21]. There were also some other genes, which were regulated in haGSCs such as* JARID2*/*JUMONJI*,* DNMT3A*, and* HOOK1*. It was shown by Shen et al. [22] that the JARID2/JUMONJI is a DNA-binding protein that functions as a transcriptional repressor and modulates POLYCOMB activity and self-renewal versus differentiation of stem cells during embryonic development. This protein facilitates the recruitment of the PRC2 complex to target genes [33]. These authors have also shown that* JARID2* regulates the binding of* POLYCOMB* repressive complex 2 to target genes in ESCs and is therefore responsible for the proper differentiation of ESCs and normal development. It is interesting that epigenetically regulated gene* DNMT3A* was also regulated in haGSCs. In general, the mammalian cells can epigenetically modify their genome by DNA methylation. The protein DNMT3A functions as a* de novo* methyltransferase and was found to be highly expressed in mitotically quiescent human fetal spermatogonia [25, 26]. In addition to the established spermatogonial markers, haGSCs were found to express the ES cell-associated gene* HOOK1*, which is a cytosolic protein attached to the microtubules that mediates the binding to cell organelles. The HOOK1 protein was found to be present at high levels in human testes [27].

There were also some other genes related to pluripotency and ES cells which were regulated in haGSCs, such as* ACTIVIN A* and* ACTIVIN receptors IIA* and* IIB*,* IGFR2BP3*, and* IGF2*. The data from the literature indicate that* ACTIVIN A* and* ACTIVIN receptors IIA* and* IIB* may be involved in the regulation of germ cell proliferation in the human ovary during the period leading up to primordial follicle formation. The insulin growth factors are known to have a key role in maintaining the status of pluripotency [28]. For example, the gene* IGFR2BP3,* which encodes a member of the IGF-II mRNA-binding protein (IMP) family, was also upregulated in haGSCs. The encoded protein binds to the 5′ UTR of the insulin-like growth factor 2 (IGF2) mRNA and thereby regulates the IGF2 translation.

Also* REX1 *(*ZFP42*) is another gene whose expression was regulated in haGSCs and is known to be closely associated with pluripotency/multipotency in both mouse and human embryonic stem cells [30]. It was demonstrated that the* REX1* (*ZFP42*) null mice show impaired testicular function, abnormal testis morphology, and aberrant gene expression. Also* BRD7,* a novel PBAF-specific SWI/SNF subunit which was expressed in haGSCs, is known to be required for target gene activation and repression in embryonic stem cells [29]. There were also some reprogramming-related genes which were expressed in haGSCs. Kuo et al. [34] documented the novel role of* miR-302/367* in reprogramming, a process which is normally involved in the early embryonic development and embryonic stem cell formation.

According to the microarray analysis in this study most of ESC-associated genes (including* PROMININ1* and* MYCN*) which were upregulated in haGSCs were also upregulated in enriched population of spermatogonia cultured* in vitro*, but not in hFibs. The correlation analysis of the microarray data showed that haGSCs are more closely related to spermatogonia. This might be related to their cellular origin.

From the single cell population, it became obvious that, under the conditions which we employed to select and maintain long-term culture haGSCs, the cells are a heterogeneous population of cells where most of the cells possess a residual expression of germ cell genes (*TSPY*,* CD9*,* GFRα1*, and* STELLA*), but only 25% of the cells express the core pluripotency genes* OCT4*,* NANOG*, and* SOX2*.

Recently, there were more reports on the presence of MSCs in adult human testicles [10–12]. In contrast to them [10–12], we found that most of the genes, proposed to be expressed in testicular MSCs, were not expressed in the haGSCs presented in this study. In the studies of Mizrak et al. [5], Chikhovskaya et al. [7], and Gonzalez et al. [8], different populations of testicular stem cells might be isolated from the tissue and cultured* in vitro*. It is not excluded that there are different types of stem cells present in adult human testicles, which might interact and reflect the complexity of this reproductive organ.

In conclusion, the molecular analysis in this study confirmed that haGSCs were generated from the enriched population of CD49f MACS and matrix-selected spermatogonia. During the long-term culture* in vitro*, they reexpressed some genes related to developing germ cells in culture, which might be otherwise blocked by their natural niche, testicular tubules, in adult human testicles. The haGSCs are a heterogeneous population of cells that are different from hFibs or MSCs and express a degree of pluripotency. The further research is needed to optimize the culture condition to avoid the molecular block, which prevents the haGSCs from becoming fully molecular pluripotent stem cells.

## 4. Conclusions

During the cell culture, haGSCs originate in the enriched population of CD49f MACS and matrix-selected spermatogonia, but never in the negatively selected fraction or from patients without spermatogonia [1]. The haGSC colonies were easily distinguishable from hFibs and resembled the early hESC colonies characterized by central cluster with outgrowing “epithelial”-like cells. By single-cell Fluidigm analysis it was found that haGSCs were quite distinct from hFibs in terms of the expression of germ- and pluripotency-associated genes. Only a minority of outliner hESCs and haGSCs shared some similarities with hFibs, but the majority of them did not. It also became clear that haGSC colonies were heterogeneous, displaying more or less similarities to a state of pluripotency. Also in the microarray study different haGSC colonies were confirmed to be relatively heterogeneous in terms of the expression of germ- and pluripotency-associated genes. When analysing the whole transcriptome and the high variance genes in haGSCs in comparison with hESCs and hFibs, it was found that the haGSCs separated from hFibs and represent a specific population of cells.

## 5. Material and Methods

### 5.1. Testicular Tissue and Experimental Design

This study was conducted from October 2009 to September 2012 using testicular material from 5 adult men, patients (P157, 159, 171, 239, and 240) with different medical background. The detailed information on patient's data is provided in Supplementary Table 1. All experiments with human material conducted here were approved by the local ethics councils (University Hospitals of Tübingen and Heidelberg) and informed written consent was obtained from all the human subjects. Age of the patients ranged from 23 to 67 years. Healthy donated tissue included heterogeneous material from patients with different medical background including orchiectomies as part of a reassignment surgery of transsexual patients after hormone therapy (1), orchiectomies of healthy testis in case of penis carcinoma and prostate cancer (2), and biopsies of “healthy” (nonmalignant) peritumoral testicular tissue from patients with seminoma (3). Histopathological examinations of the testicular tissue used in this study were conducted by experts at the Department of Pathology (University Clinic, Tübingen) in routine diagnostics and in case of cancer with more cancer specific diagnostics.

In this study short-term (<2 weeks after matrix selection) SSC cultures and long-term (>2 months, up to 6 months) haGSC cultures from testicular tissues of all 5 men were analyzed on gene expression profile to evaluate the character of testicular adult stem cells. The experimental design of this study can be seen in Supplementary Table 2. The single cells from the different groups of cells were first analyzed on gene expression profile by Biomark Real-Time quantitative PCR (qPCR) system (Fluidigm), followed by microarray analysis in comparison with hESCs and hFibs. The selected group of genes from microarray analysis was validated by Biomark Real-Time quantitative PCR (qPCR) system (Fluidigm). We mainly focused on pluripotency-, germ cell-, fibroblast- and mesenchymal stem cell-associated genes.

### 5.2. Selection and Cultivation of haGSCs

After removing of the tunica albuginea, the obtained human testicular tissues were mechanically disrupted to dissociate the tubules. In each sample, the dissociated tubules were enzymatically digested with 750 U/mL collagenase type IV (Sigma), 0.25 mg/mL dispase II (Roche), and 5 *μ*g/mL DNase in HBSS buffer with Ca^++^ and Mg^++^ (PAA) for 30 minutes at 37°C, with gentle mixing, to obtain a single-cell suspension. Then the digestion was stopped with 10% ES cell-qualified FBS. The cell suspension was passed through a 100 *μ*m cell strainer and centrifuged for 15 minutes at 1000 rpm. The supernatant was removed and the pellet was washed with HBSS buffer with Ca^++^ and Mg^++^. After washing, the cells (approximately 2 × 10^5^ cells per cm^2^) were plated into culture dishes (*d* = 10 cm), coated with 0.2% gelatin (Sigma), in hGSC (human germ stem cell) medium consisting of StemPro hESC medium, 1% N2-supplement (Invitrogen), 6 mg/mL D+ glucose (Sigma), 5 *μ*g/mL bovine serum albumin (Sigma), 1% L-glutamine (PAA), 100 *μ*M *β*-mercaptoethanol (Invitrogen), 1% penicillin/streptomycin (PAA), 1% MEM vitamins (PAA), 1% nonessential amino acids (PAA), 30 ng/mL estradiol (Sigma), 60 ng/mL progesterone (Sigma), 20 ng/mL epidermal growth factor (EGF; Sigma), 10 ng/mL basic fibroblast growth factor (FGF; Sigma), 8 ng/mL glial-derived neurotrophic factor (GDNF; Sigma), 100 U/mL human LIF (Millipore), 1% ES cell qualified FBS, 100 *μ*g/mL ascorbic acid (Sigma), 30 *μ*g/mL pyruvic acid (Sigma), and 1 *μ*L/mL DL-lactic acid (Sigma). In this culture medium, the cells were incubated in a CO_2_-incubator for 96 hours at 37°C and 5% CO_2_ in air. After 72 hours the half volume of culture medium was replaced with fresh culture medium of the same volume and the cells were further cultured for 4 days. On day 7 the culture medium was carefully removed and the testis cell culture was gently rinsed with 5 mL DMEM/F12 culture medium with L-glutamine (PAA) per plate to harvest the germ cells bound to the monolayer of adherent somatic cells attached to the dish bottom. This procedure was repeated by pipetting 5 mL of DMEM/F12 culture medium. The cell suspension pooled from 5 culture dishes per tissue sample was centrifuged for 5 minutes at 1000 rpm. The pellet was resuspended in 10 mL of MACS buffer and centrifuged again for 5 minutes and the cells were further purified with MACS separation (Miltenyi), CD49f-FITC (*α*_6_-integrin; AbD Serotec), and anti-FITC beads (Miltenyi). After MACS separation, cells were transferred to dishes coated with collagen I (5 *μ*g/cm^2^, Becton & Dickinson) and incubated at 37°C for 4 h. Cells that did not bind to collagen I dishes (Col_NB_ cells) were harvested and pelleted at 1000 rpm. The Col_NB_ cells were suspended in medium and plated at 0.5–1 × 10^6^ cells per mL per well in 12-well plates precoated with laminin (4.4 *μ*g/cm^2^, Sigma). The plated Col_NB_ cells were incubated for 45 min at 37°C and unbound cells (Col_NB_/Lam_NB_ cells) were removed from bound cells (Lam_B_ cells) by pipetting and were discarded. The Lam_B_ cells were rinsed twice with 1 mL media. The Lam_B_ cells then were harvested by gentle pipetting and were plated onto a 12-well plate with hGSC culture medium, on irradiated CF-1 feeder layer. A half volume of culture medium was removed every 2-3 days and replaced with fresh hGSC culture medium. Under these conditions, the spermatogonia heterogeneously proliferated. The best cell cultures were split 1 : 2 every two to three weeks. It was important not to dilute the cells too much and to keep the appropriate cell number in the wells all the time.

### 5.3. Cultivation of Human Fibroblasts

The human fibroblasts were obtained from the dermis of the scrotum and a primary cell line was generated in DMEM high glucose, 10% FBS Superior (Biochrom), 200 *μ*M L-glutamine (PAA), 1% nonessential amino acids (PAA), and 100 mM *β*-mercaptoethanol (Invitrogen).

### 5.4. Cultivation of hESCs

The H1 human ES cell line from the National Stem Cell Bank were cultured, respectively, according to the protocols from WiCell on CF1 Feeder in DMEM/F12 with L-glutamine (PAA), 20% knockout serum replacement (Invitrogen), 300 *μ*M L-glutamine (PAA), 1% nonessential amino acids (PAA), 100 mM *β*-mercaptoethanol (Invitrogen), 1 mM HEPES, and 4 ng/mL basic fibroblast growth factor (FGF, Sigma).

### 5.5. Collection of Single Cells from the Population of Enriched Spermatogonia (hSSCs) with Micromanipulation System

In each sample, the spermatogonial cells were rinsed with the culture medium to remove the spermatogonia from the attached monolayer of somatic cells or feeder layer in a culture dish. After gentle resuspension, the cells were transferred to a single cell suspension into the top of small culture dish (*d* = 3.5 cm). The top of dish was placed onto a prewarmed (37°C) working platform of a Zeiss inverted microscope with the micromanipulation system. At magnification 20x, the cells were collected step by step by a micromanipulation pipette. The typical morphology of short-term cultured spermatogonia was clearly observed. This was primarily based on their round shape, diameter of approximately 6–12 *μ*m, and high nucleus-to-cytoplasm ratio, which could be observed by a clear small shining cytoplasmic ring between the round nucleus and the outer cell membrane.

### 5.6. Collection of Single Cells from Enzymatically Degraded Typical haGSC Colonies, hESCs, and hFibs with a Micromanipulation System

In order to characterize the single cells in the haGSC colony, we enzymatically degraded a typical haGSC and hESC colony or confluent growing hFibs to a single cell level and manually selected individual cells one by one (24 hFibs, 24 hESCs, and 48 haGSCs) with a micromanipulation system for single cell gene expression profiling. With this technique, we aimed to provide information about single cell profiles of important germ- and pluripotency-associated genes in these cells and the homo-/heterogeneity of the selected cells from a typical haGSC colony and to culture further those colonies with the “best” germ- and pluripotency-associated gene expression profile. Single cells per sample probe were collected for Fluidigm analysis and 200 cells per probe for microarray analysis and also for validation of selected pluripotency associated genes by Fluidigm analysis. After collection, the cells were directly transferred into 6.5 *μ*L of cells direct buffer for Fluidigm or 10 *μ*L RNA direct lyses buffer for microarray analysis.

### 5.7. Gene Expression Analyses by Fluidigm Biomark System

Gene expression analysis of single cells and 200 cells was performed using the Biomark Real-Time quantitative PCR (qPCR) system (Fluidigm) in comparison with hESCs (positive control) and human testis hFibs (F161; negative control). In all cell samples the expression of the following genes was analyzed by Taqman assays: germ cell-specific genes* TSPYL*,* DDX4* (*VASA*),* DAZL*,* ZBTB16* (*PLZF*),* DPPA3* (*STELLA*),* CD9*,* NANOS*,* UTF1*,* GFRα1*,* GPR125*,* REX1*,* KIT*,* KIT LG*,* LIFR*,* STAT3*, pluripotency-associated genes* POU5F1 (OCT4)A*,* POU5F1 (OCT4)B*,* LIN28*,* NANOG*,* SOX2*,* GDF3*,* KLF4*,* MYC*,* TDGF1*,* TERT*,* DNMT3B*,* DNMT1*,* CDH1*,* LIN28B*,* OCT4B*, and the housekeeping genes 18SRNA,* CTNNB1*,* HNBS*, and* GAPDH*.

According to genes upregulated in haGSC clone 157-23_P5 in the microarray experiment, the following further assays were selected:* SALL4* (sal-like 4),* SALL2* (sal-like 2),* PROM1* (prominin 1),* EPCAM* (epithelial cell adhesion molecule),* GATA4* (GATA binding protein 4),* HOOK1* (hook homolog 1),* L1TD1* (LINE-1 type transposase domain containing 1),* JARID2* (JUMONJ),* IGFR2 BP3* (insulin-like growth factor 2 mRNA binding protein 3),* REX1* (zinc finger protein 462), and* ACVAR1B* (activin receptor-like kinase 4).

The inventoried TaqMan assays (Applied Biosystem) were pooled to a final concentration of 0.2x for each of the assays. Cells to be analyzed were harvested directly into 9 *μ*L RT-PreAmp Master Mix consisting of 5.0 *μ*L CellsDirect 2x Reaction Mix (Invitrogen), 2.5 *μ*L 0.2x assay pool, 0.2 *μ*L RT/Taq Superscript III (Invitrogen), and 1.3 *μ*L TE buffer. The harvested cells were immediately frozen and stored at −80°C. Cell lysis and sequence-specific reverse transcription were performed at 50°C for 15 min. The reverse transcriptase was inactivated by heating to 95°C for 2 minutes. In the same tube, cDNA subsequently underwent limited sequence-specific amplification by denaturing at 95°C for 15 seconds and 14 cycle-annealing and amplification at 60°C for 4 minutes. These preamplified products were 5-fold diluted prior to analysis with Universal PCR Master Mix and inventoried TaqMan gene expression assays (ABI) in 96.96 Dynamic Arrays on a Biomark system. Each sample was analyzed in two technical replicates.

### 5.8. GenEx Statistical Analysis

Ct values obtained from the Biomark system were transferred to the GenEx software (MultiD) for analysis. Missing data in the Biomark system were assigned a Ct of 999 by the instrument software. These were removed in GenEx. Also Ct's larger than a cutoff of 25 were removed, since high Ct's in the Biomark 96 × 96 microfluidic card were expected to be false positives due to baseline drift or formation of aberrant products and since a sample with a single template molecule is expected to generate a lower Ct. The effect of setting cutoff to 25 was tested by repeating the analysis with a slightly different cutoff and was found to have negligible effect on the analysis results. Technical repeats were then averaged and any remaining missing data were replaced by the highest Cq measured + an offset of 1 for each gene separately. Managing missing data is primarily required for downstream multivariate classification of the data. An offset of 1 corresponds to assigning a concentration to the samples with off-scale Cq values, that is, half of the lowest concentration measured for a truly positive sample. The magnitude of the offset does not influence *P* values calculated with nonparametric methods, which were preferred when there were off-scale data, but it has small influence on *P* values calculated by *t*-test and on multivariate classification. In essence, the offset tunes and the weight of the off-scale measurement compared to the positive reading; larger offset gives higher weight to the off-scale measurement. We tested the importance of the offset by repeating the analysis using a higher offset up to +4, which corresponds to a concentration of 6% of a truly positive sample, and found negligible effect on the multivariate results. Linear quantities were calculated relative to the sample having lowest expression and data were then converted to log_2_⁡ scale for analysis. Because of the very large and uncorrelated cell-to-cell variation of genes' expressions normalization to the housekeeping genes is not meaningful. Instead, expression levels were presented “per 50-cell” average expression of the genes in different groups was calculated including .95% confidence interval and groups were compared using 1-way ANOVA (Tukey-Kramer pairwise comparison) and unpaired 2-tailed *t*-test. Expression of genes with multiple off-scale readings was compared with nonparametric Mann-Whitney test. For multivariate analysis to classify the samples based on the combined expression of all the genes data were either mean centred, that is, subtracting the average expression of each gene, or autoscaled, which is mean centre data also divided by the standard deviation (so called *z*-score). Autoscaling gives all the genes equal weight in the classification algorithms making them equally essential. Hierarchical clustering (Ward's algorithm, Euclidean distance measure) including heatmap and principal component analysis (PCA) were performed.

### 5.9. Microarray Analysis

The total RNA isolated from short-term spermatogonia and long-term haGSC cultures, hESC line H1 (positive control), and testicular fibroblasts (hFibs; negative control) was prepared using the RNeasy Mini Kit (Qiagen), followed by an amplification step with MessageAmp aRNA Kit (Ambion). In each sample, 200 cells were collected per probe with the micromanipulation system and transferred directly into 10 *μ*L of RNA direct lysis solution and stored at −80°C. Samples were analysed at the microarray facility of the University of Tübingen Hospital, Germany. Gene expression analysis was performed using the Human U133 + 2.0 Genome oligonucleotide array (Affymetrix). The raw data (CEL-files) was provided to the MicroDiscovery GmbH, Berlin, Germany, for normalization and biostatistical analysis.

### 5.10. Microarray Data Normalisation and Analyses

Microarray data was imported into the R Statistical Environment version 2.12.1 (2010-12-16). Data condensation was performed using Bioconductor package affy version 1.28.0. The condensation criteria were as follows: bg.correct = FALSE, normalize = FALSE, pmcorrect.method = “pmonly,” and summary.method = “medianpolish.” Additional normalization was performed between samples using multi-lowess algorithm, a multidimensional extension of lowess normalization strategy [15]. The data were analyzed in terms of sample to sample relations, high variance genes, predefined gene sets for germ-, pluripotency-, fibroblast-, and MSC-associated genes from the literature, and extended profile search. A proportion of data was transferred into the IPA Ingenuity program to evaluate the gene functions and pathways. Additional data are provided in the Supplemental Methods section.

### 5.11. Antibodies and Staining

The following primary antibodies were used as stem cell markers: mouse monoclonal anti-CD9 (R&D System, Stem cell marker kit, SC009), mouse monoclonal anti-CD24 (Abcam, ab31622), rabbit polyclonal anti-OCT4 (Abcam, ab19857), and rabbit polyclonal anti-nanog (Abcam, ab21624). For the staining of germ cells, the following markers were used: rabbit polyclonal anti-VASA (Abcam, ab13840), mouse monoclonal anti-UTF1 (Chemicon, MAB4337), rabbit polyclonal anti-TSPYL2 (Proteintech, 12087-2-AP), rat monoclonal anti-STELLA (R&D Systems, MAB2566), and goat polyclonal anti-GFR*α*1 (R&D Systems, AF714).

Alexa Fluor-488-conjugated goat anti-mouse IgG, Alexa Fluor-488-conjugated goat anti-rabbit IgG, Alexa Fluor-488-conjugated donkey anti-goat IgG, Alexa Fluor-546 goat anti-mouse IgG, Alexa Fluor-546 goat anti-rabbit IgG, and Alexa Fluor-546-conjugated goat anti-rat IgG were used as secondary antibodies. Nuclear costaining was performed for stem cell markers with DAPI and for germ cell markers with Hoechst.

## Supplementary Material

In the supplements, 4 Figures including detailed bar plots of Fluidigm real-time PCRs with germ-, and pluripotency-related gene expression profiling of hFibs, hESCs, haGSCs are shown, followed by more heat maps, PCAs displaying various aspects of microarray analysis and real-time PCRs validating the microarray experiments.
Furthermore Suppl. Tables with patient's data, experimental design and the most up-regulated genes in the different comparisons between hFibs, hESC and haGSCs according to the Fluidigm real-time PCRs are provided. Furthermore tables with functional annotations are shown.
In the Suppl. Methods section more details about data normalization for the microarray analysis and GenEX analysis for Fluidigm real-time PCR data are provided.
