# Supplementary material for: Expression of Genes Related to Germ Cell Lineage and Pluripotency in Single Cells and Colonies of Human Adult Germ Stem Cells
Source: Stem Cells Int. 2015 Nov 8;2016:8582526. doi: 10.1155/2016/8582526 (PMC4655073; doi:10.1155/2016/8582526)
Supplement: Supplementary file 1 — In the supplements, 4 Figures including detailed bar plots of Fluidigm real-time PCRs with germ-, and pluripotency-related gene expression profiling of hFibs, hESCs, haGSCs are shown, followed by more heat maps, PCAs displaying various aspects of microarray analysis and real-time PCRs validating the microarray experiments. Furthermore Suppl. Tables with patient's data, experimental design and the most up-regulated genes in the different comparisons between hFibs, hESC and haGSCs according to the Fluidigm real-time PCRs are provided. Furthermore tables with functional annotations are shown. In the Suppl. Methods section more details about data normalization for the microarray analysis and GenEX analysis for Fluidigm real-time PCR data are provided. [file 8582526.f1.docx]

**Suppl. Figure 1**


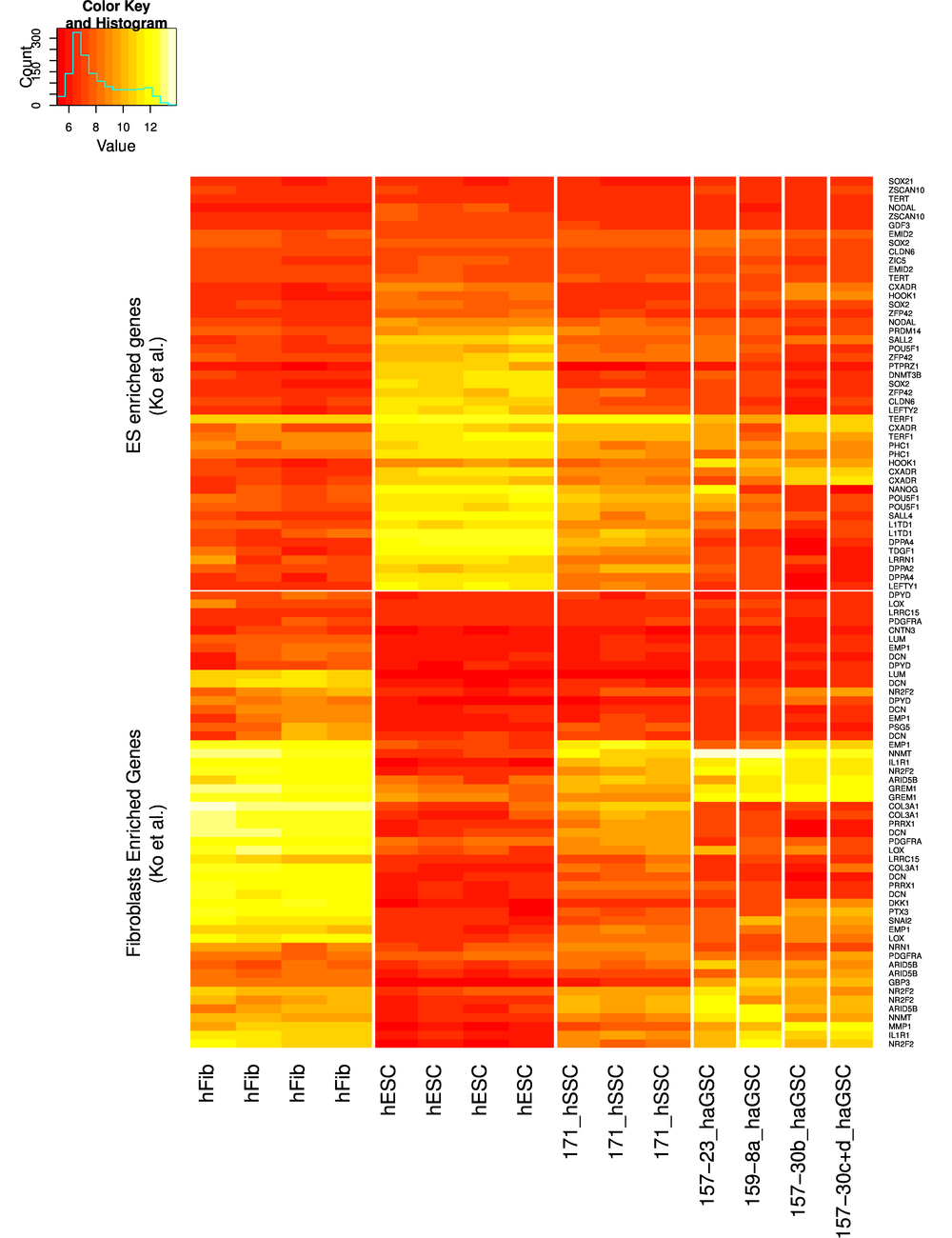


**Suppl. Figure 2**


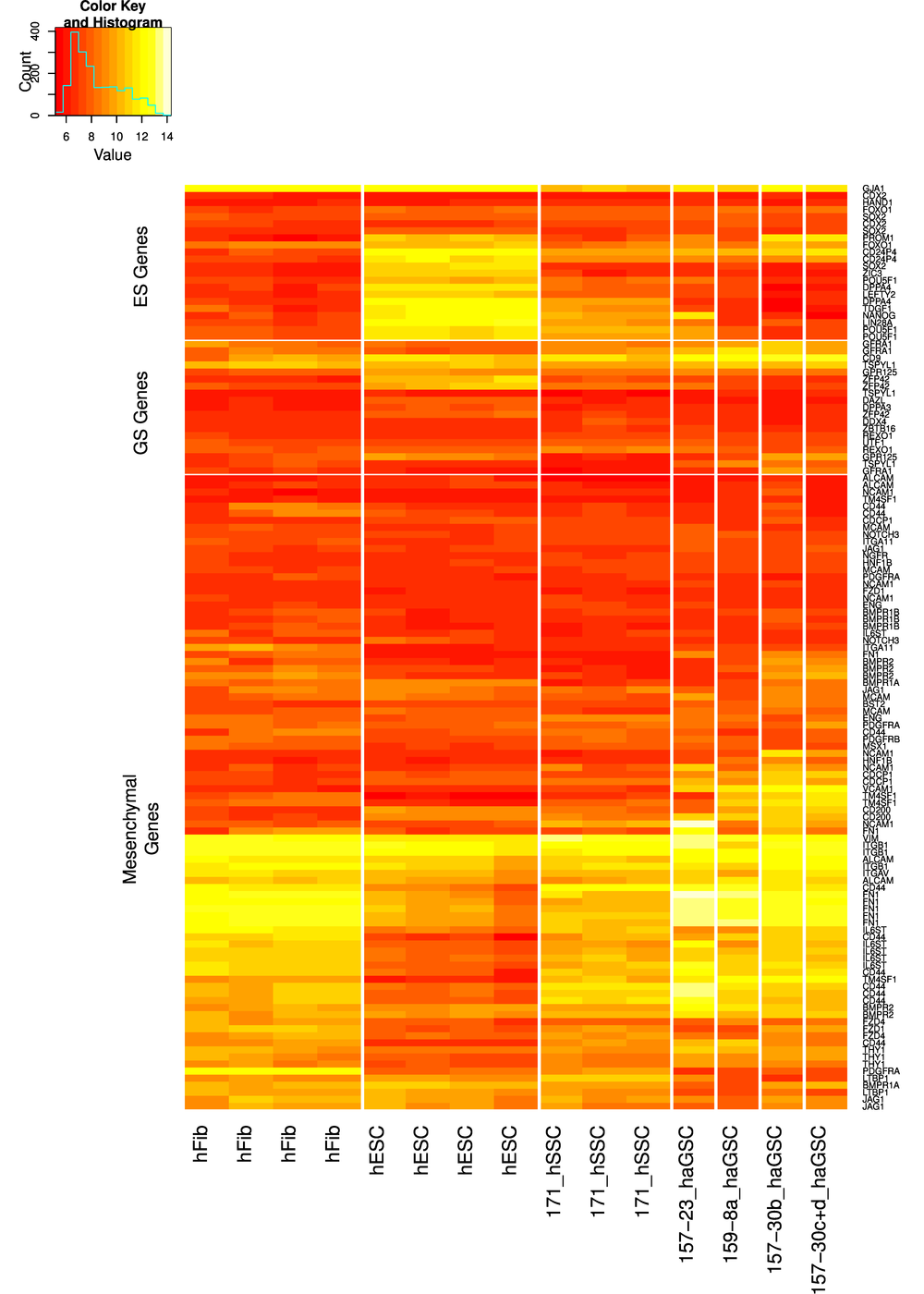


**Suppl. Figure3**


**Suppl. Figure 4a**

**Suppl. Figure 4b**

**Suppl. Tables:**

**Suppl. Table 1**: Overview of the patients with age, diagnostics and histo-pathological testicular tissue characterization.

**Suppl. Table 2.** Experimental design in terms of patients, culture and analyses.

|  | **SHORT-TERM CULTURES**  **(SSCs)** | | **LONG-TERM CULTURES**  **(haGSCs)** | |
| --- | --- | --- | --- | --- |
| **Patient** | **Duration of culture** | **Method of analysis** | **Duration of culture** | **Method of analysis** |
| **157** |  |  | One culture  (3 months) | Single-cell Fluidigm analyses  (pilot study) |
|  |  |  | Two independent cultures  (6 months) | Microarrays |
| **159** |  |  | Two independent cultures  (6 months) | Microarrays |
| **171** | Four independent cultures  (< 2 week) | Microarrays |  |  |
| **239** |  |  | One culture  (5 months) | Fluidigm analyses  (validation  of microarrays) |
| **240** |  |  | One culture  (5 months) | Fluidigm analyses, IMH (validation  of microarrays) |

**Suppl. Table 3: t-tests are presented showing fold change and difference in log scale and the corresponsing p-values comparing hFibs, hESCs and haGSCs. Only data with significant P-Value are shown.**

**(A) (hFibs) vs (haGSCs)**

| **Gene** | **Fold change** | **Difference (log scale)** | **P-Value** |
| --- | --- | --- | --- |
| CD9 | -474,04117 | -8,88887 | 1,00E-08 |
| NANOS | -23,48087 | -4,55341 | 2,70E-08 |
| GFRa1 | -49,80956 | -5,63835 | 8,43E-07 |
| KLF4 | 44,78063 | 5,4848 | 1,48E-06 |
| STELLA | -11,00012 | -3,45945 | 8,22E-06 |
| TDGF1 | -13,31284 | -3,73475 | 2,52E-05 |
| DNMT3B | -7,23026 | -2,85405 | 3,40E-05 |
| TSPY | -11,86544 | -3,56869 | 0,000266397 |
| NANOG | -11,89511 | -3,5723 | 0,000475066 |
| STAT3 | -4,40327 | -2,13858 | 0,000627668 |
| Oct4b | -6,08666 | -2,60565 | 0,000680058 |
| LIN28 | -14,97538 | -3,90452 | 0,001087502 |
| GPR125 | -18,79816 | -4,23252 | 0,006241753 |
| SOX2 | -3,25128 | -1,70101 | 0,008042156 |
| Oct4a | -4,67693 | -2,22556 | 0,008074453 |

**(B) (hFibs) vs (haGSC I)**

| **Gene** | **Fold change** | **Difference (log scale)** | **P-Value** |
| --- | --- | --- | --- |
| STELLA | -31,6517 | -4,98421 | 1,00E-08 |
| TDGF1 | -61,38079 | -5,93972 | 1,00E-08 |
| CD9 | -1241,88537 | -10,27832 | 1,00E-08 |
| NANOS | -109,54474 | -6,77538 | 1,00E-08 |
| GFRa1 | -244,57269 | -7,93412 | 1,00E-08 |
| DNMT3B | -17,59628 | -4,1372 | 2,90E-08 |
| NANOG | -44,96785 | -5,49082 | 5,88E-07 |
| GPR125 | -270,88731 | -8,08155 | 1,38E-06 |
| LIN28 | -73,95446 | -6,20857 | 2,37E-06 |
| Oct4b | -12,52988 | -3,6473 | 3,57E-06 |
| STAT3 | -12,64777 | -3,66081 | 1,05E-05 |
| SOX2 | -6,76542 | -2,75818 | 2,53E-05 |
| TSPY | -36,98054 | -5,20869 | 5,96E-05 |
| OCT4a | -10,41906 | -3,38115 | 0,001314653 |
| KLF4 | 25,05874 | 4,64724 | 0,002363239 |
| MYC | -5,25073 | -2,39252 | 0,002660463 |
| DNMT1 neu | -3,65654 | -1,87048 | 0,006203616 |

**(C) (hFibs) vs (hESCs cells)**

| **Gene** | **Fold change** | **Difference (log scale)** | **P-Value** |
| --- | --- | --- | --- |
| Oct4b | -36,5788 | -5,19294 | 1,00E-08 |
| LIN28 | -6148,86468 | -12,5861 | 1,00E-08 |
| NANOG | -5983,82384 | -12,54685 | 1,00E-08 |
| SOX2 | -922,59319 | -9,84955 | 1,00E-08 |
| TDGF1 | -1036,89654 | -10,01806 | 1,00E-08 |
| DNMT3B | -998,0005 | -9,9629 | 1,00E-08 |
| OCT4a | -183,43545 | -7,51913 | 1,00E-08 |
| STELLA | -19,69353 | -4,29965 | 1,15E-07 |
| CD9 | -12,24147 | -3,6137 | 2,39E-05 |
| DNMT1 neu | -7,39213 | -2,88599 | 2,48E-05 |
| NANOS | -4,41804 | -2,14341 | 0,001058595 |
| GPR125 | -24,99475 | -4,64355 | 0,004517463 |
| GFRa1 | -7,48612 | -2,90422 | 0,015508495 |
|  |  |  |  |

**(D) (hESCs) vs (hGSCs I)**

| **Gene** | **Fold change** | **Difference (log scale)** | **P-Value** |
| --- | --- | --- | --- |
| CD9 | -101,44904 | -6,66461 | 1,00E-08 |
| SOX2 | 136,36905 | 7,09137 | 1,00E-08 |
| NANOS | -24,79488 | -4,63197 | 6,90E-08 |
| DNMT3B | 56,71656 | 5,8257 | 1,16E-07 |
| NANOG | 133,06893 | 7,05603 | 1,49E-07 |
| CDH1 | 76,55947 | 6,25851 | 2,70E-07 |
| STAT3 | -16,06906 | -4,00621 | 3,57E-06 |
| OCT4a | 17,60575 | 4,13797 | 1,65E-05 |
| Lin28 | 83,14394 | 6,37754 | 1,85E-05 |
| GFRa1 | -32,67016 | -5,0299 | 0,000214764 |
| UTF1 | 9,3808 | 3,22971 | 0,000347909 |
| TSPY | -13,53306 | -3,75842 | 0,000683482 |
| TDGF1 | 16,89285 | 4,07834 | 0,002076236 |
| GPR125 | -10,83777 | -3,438 | 0,032079674 |
| MYC | -2,99397 | -1,58206 | 0,045726942 |

|  | **GOID** | **GOTERMS** | **pValue** | **pFDR** |
| --- | --- | --- | --- | --- |
| haGSC | GO:0018262 | isopeptide cross-linking | 2,30E-08 | 9,35E-06 |
| haGSC | GO:0018199 | peptidyl-glutamine modification | 1,15E-07 | 2,36E-05 |
| haGSC | GO:0032103 | positive regulation of response to external stimulus | 1,57E-07 | 2,36E-05 |
| haGSC | GO:0022610 | biological adhesion | 2,14E-07 | 2,36E-05 |
| haGSC | GO:0060605 | tube lumen cavitation | 4,80E-07 | 4,00E-05 |
| haGSC | GO:0007155 | cell adhesion | 6,19E-07 | 4,23E-05 |
| haGSC | GO:0009611 | response to wounding | 9,04E-07 | 5,66E-05 |
| haGSC | GO:0006909 | phagocytosis | 1,56E-06 | 8,36E-05 |
| haGSC | GO:0006952 | defense response | 2,35E-06 | 1,08E-04 |
| haGSC | GO:0033364 | mast cell secretory granule organization | 3,27E-06 | 1,08E-04 |
| hFib | GO:0044421 | extracellular region part | 5,61E-16 | 9,72E-14 |
| hFib | GO:0005576 | extracellular region | 5,67E-16 | 9,72E-14 |
| hFib | GO:0005581 | collagen | 6,97E-16 | 9,72E-14 |
| hFib | GO:0031012 | extracellular matrix | 2,32E-15 | 1,03E-13 |
| hFib | GO:0044420 | extracellular matrix part | 4,29E-13 | 1,65E-11 |
| hFib | GO:0005578 | proteinaceous extracellular matrix | 4,75E-13 | 1,65E-11 |
| hFib | GO:0006029 | proteoglycan metabolic process | 2,64E-11 | 7,46E-10 |
| hFib | GO:0030934 | anchoring collagen | 1,24E-10 | 3,10E-09 |
| hFib | GO:0030247 | polysaccharide binding | 3,34E-10 | 7,58E-09 |
| hFib | GO:0001871 | pattern binding | 5,79E-10 | 1,18E-08 |
| hESC | GO:0048598 | embryonic morphogenesis | 9,47E-07 | 1,86E-04 |
| hESC | GO:0010586 | miRNA metabolic process | 1,21E-06 | 1,86E-04 |
| hESC | GO:0009790 | embryo development | 2,39E-06 | 1,86E-04 |
| hESC | GO:0010557 | positive regulation of macromolecule biosynthetic process | 3,04E-06 | 1,86E-04 |
| hESC | GO:0031328 | positive regulation of cellular biosynthetic process | 4,20E-06 | 1,86E-04 |
| hESC | GO:0009891 | positive regulation of biosynthetic process | 4,78E-06 | 1,86E-04 |
| hESC | GO:0051254 | positive regulation of RNA metabolic process | 1,15E-05 | 3,48E-04 |
| hESC | GO:0048646 | anatomical structure formation involved in morphogenesis | 1,24E-05 | 3,48E-04 |
| hESC | GO:0045941 | positive regulation of transcription | 1,29E-05 | 3,48E-04 |
| hESC | GO:0010628 | positive regulation of gene expression | 1,32E-05 | 3,48E-04 |

**Suppl. Table 2:** Functional Annotation with Gene Ontology terms of the 3 classes of genes shown in Fig. 6 with high expression in hFibs (F161), hESCs and haGSC samples.

P-values and pFDR values are calculated with gossip [ref]. The analyses were performed independently for each gene group.

**Suppl. Table 3:** Functional Annotation with Gene Ontology terms of genes derived from sectors II, IIX, IV and VI in Figure 6A. P-values and pFDR values are calculated with gossip [ref]. The analyses were performed independently for the genes of each sector.

**Suppl. Methods**

**Pre-Processing of Microarray data**

Data condensation was performed with Bioconductor package affy-1.28.0 and R-

2.12.1. Condensation was performed without background correction

and without normalization (bg.correct = FALSE, normalize = FALSE). Furthermore

only perfect matching probes were considered and medianpolish was used for summary of the probes (pmcorrect.method = ’pmonly’, summary.method = ’medianpolish’).

Raw data in this manuscript are the result of the condensation process and are

typically presented as *log*2 intensities.

**Normalization**

The data was normalized using multi lowess normalization. Multi Lowess effectively reduces systematic errors keeping a maximum of the original signal. Indeed, multi lowess normalization successfully removed the systematic bias since the median differences have vanished after normalization. The minimal sample correlation increased from 0.47 without normalization to 0.71 after normalization. The average sample correlation increased from 0.75 without normalization to 0.81 after normalization. The pair-wise scatter plot after normalization is shown in Suppl. Methods Fig. 1.


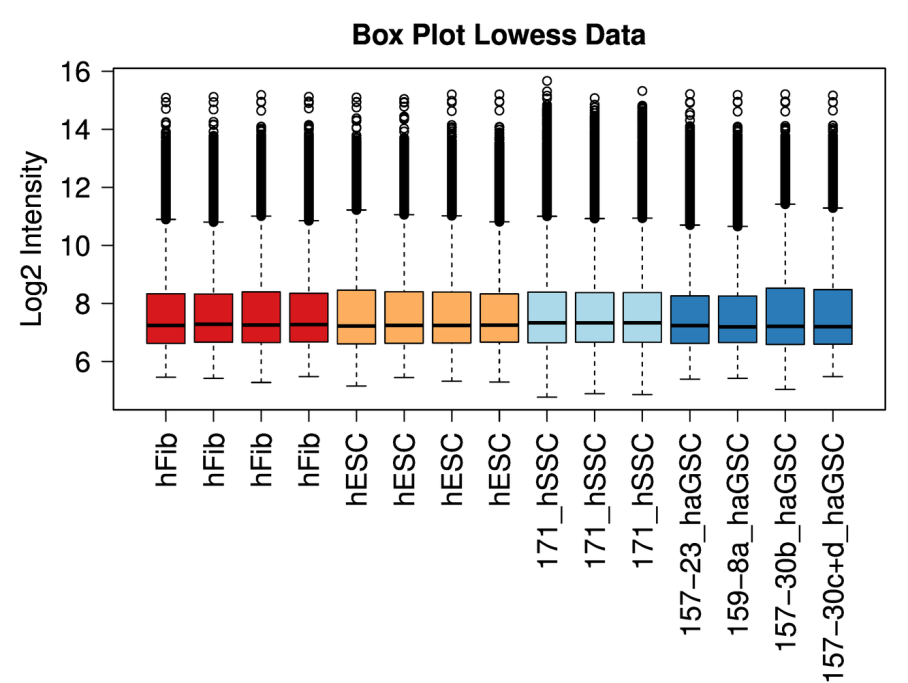


**Suppl. Methods Fig. 1**: Box plot of lowess data after outlier removal. The different cell types are colour coded. After normalization the medians of the signals are similar for each chip.


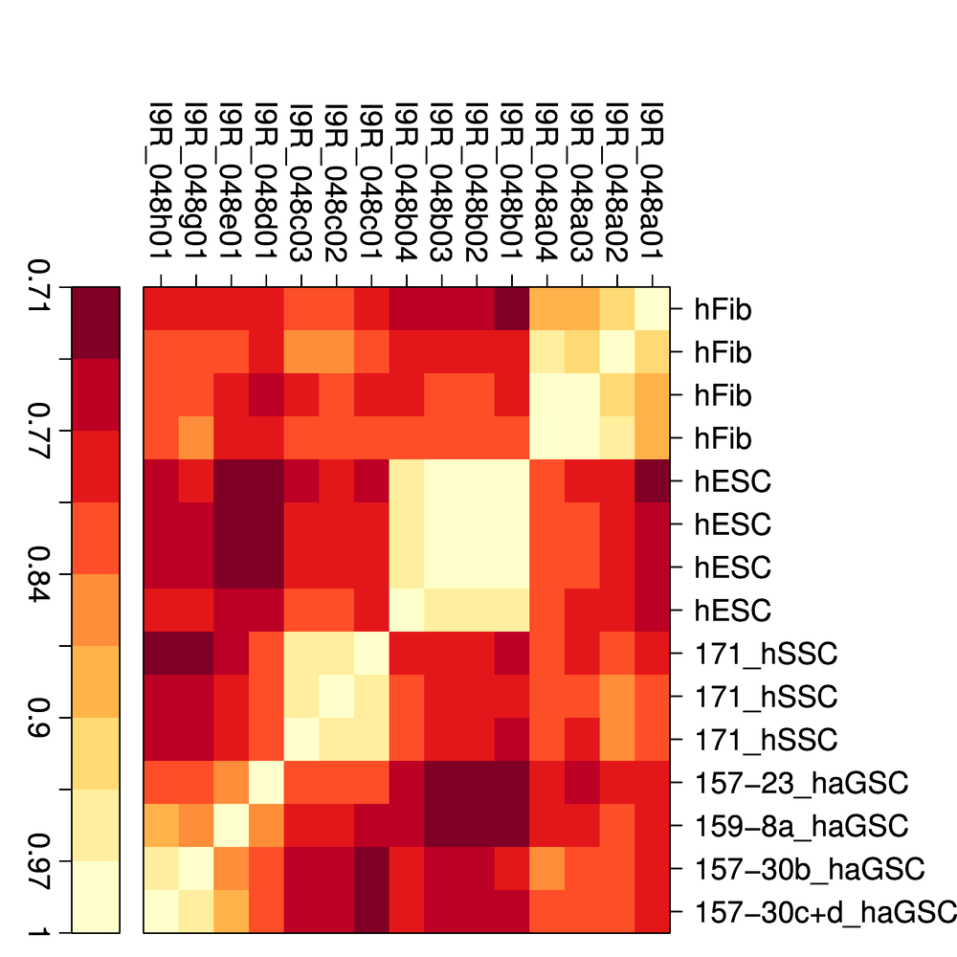


**Suppl. Methods Fig. 2**: Correlation plot of lowess normalized data after outlier removal (Correlation was calculated according to Pearson).

 **Suppl. Methods Fig. 3**: Pair-wise scatter plot of lowess normalized data.
